# Fine-mapping of a major QTL controlling angular leaf spot resistance in common bean (*Phaseolus vulgaris* L.)

**DOI:** 10.1007/s00122-015-2472-6

**Published:** 2015-03-05

**Authors:** Beat Keller, Chloe Manzanares, Carlos Jara, Juan David Lobaton, Bruno Studer, Bodo Raatz

**Affiliations:** 1 Forage Crop Genetics, Institute of Agricultural Sciences, ETH Zurich, Universitaetstrasse 2, 8092 Zurich, Switzerland; 2 Agrobiodiversity Research Area, Bean Program, CIAT Cali-Palmira, A. A. 6713, Cali, Colombia

## Abstract

*****Key message***:**

**A major QTL for angular leaf spot resistance in the common bean accession G5686 was fine-mapped to a region containing 36 candidate genes. Markers have been developed for marker-assisted selection.**

**Abstract:**

Common bean (*Phaseolus vulgaris* L.) is an important grain legume and an essential protein source for human nutrition in developing countries. Angular leaf spot (ALS) caused by the pathogen *Pseudocercospora griseola* (Sacc.) Crous and U. Braun is responsible for severe yield losses of up to 80 %. Breeding for resistant cultivars is the most ecological and economical means to control ALS and is particularly important for yield stability in low-input agriculture. Here, we report on a fine-mapping approach of a major quantitative trait locus (QTL) ALS4.1^GS, UC^ for ALS resistance in a mapping population derived from the resistant genotype G5686 and the susceptible cultivar Sprite. 180 F_3_ individuals of the mapping population were evaluated for ALS resistance and genotyped with 22 markers distributed over 11 genome regions colocating with previously reported QTL for ALS resistance. Multiple QTL analysis identified three QTL regions, including one major QTL on chromosome Pv04 at 43.7 Mbp explaining over 75 % of the observed variation for ALS resistance. Additional evaluation of 153 F_4_, 89 BC_1_F_2_ and 139 F_4_/F_5_/BC_1_F_3_ descendants with markers in the region of the major QTL delimited the region to 418 kbp harboring 36 candidate genes. Among these, 11 serine/threonine protein kinases arranged in a repetitive array constitute promising candidate genes for controlling ALS resistance. Single nucleotide polymorphism markers cosegregating with the major QTL for ALS resistance have been developed and constitute the basis for marker-assisted introgression of ALS resistance into advanced breeding germplasm of common bean.

**Electronic supplementary material:**

The online version of this article (doi:10.1007/s00122-015-2472-6) contains supplementary material, which is available to authorized users.

## Introduction

Common bean (*Phaseolus vulgaris* L.) is the most important grain legume for direct human consumption (Broughton et al. 2003), rich in protein, iron and zinc (Sathe 2002; Hemalatha et al. 2007; Martinez Meyer et al. 2013). The global production of dry and green beans is steadily increasing and has reached 44 million metric tons per year (FAOSTAT 2011). Around 111,000 years ago, common bean diverged from a common ancestor into two different gene pools: large-seeded Andean and small- to medium-seeded Mesoamerican beans (Debouck et al. 1993; Gepts 1998; Singh et al. 2002; Mamidi et al. 2013). Snap beans for green pod harvest are mainly of Andean origin (Gepts and Bliss 1985; Myers and Davis 2002). Common bean is a self-pollinating, diploid species with 11 chromosomes (2*n* = 2*x* = 22) (Mok and Mok 1976; Singh 2005), whereof 473 Mbp of DNA sequence have been assembled (Phytozome.net 2014; Schmutz et al. 2014).

Angular leaf spot (ALS) caused by the hemibiotrophic fungus *Pseudocercospora griseola* (Sacc.) Crous and U. Braun (Bassanezi et al. 2002; Crous et al. 2006) was reported to be responsible for up to 60 and 80 % yield losses in Brazil (de Jesus et al. 2001) and Colombia (Schwartz et al. 1981), respectively. *P. griseola* spores germinate on the leaf surface after 3 days of moist conditions, enter the leaf through the stomata and grow intercellularly, limited by the leaf veins resulting in an angular lesion shape (Monda et al. 2001; Willocquet et al. 2004). Infection and sporulation occurs in a broad temperature range, from 10 to 33 °C (reviewed in Allorent and Savary 2005). Following the two gene pools of common bean, Mesoamerican and Andean, the pathogen evolved in each gene pool separately (Guzman et al. 1995; Crous et al. 2006). Whereas Andean pathogens are mainly virulent on Andean beans, Mesoamerican and some Andean isolates found in Africa attack both Andeans and Mesoamerican beans (Pastor-Corrales et al. 1998; Mahuku et al. 2002). Since *P. griseola* is highly variable (Abadio et al. 2012), breeding for a broad ALS resistance involves genes of Andean and Mesoamerican origin or introduction of resistance genes from the secondary gene pool (Mahuku et al. 2003). Genetic resistance is the most efficient strategy to prevent yield loss considering ecological and economic factors (Miklas et al. 2006). However, only few resistance genes have been tagged with closely linked markers.

In Mesoamerican germplasm, the ALS resistance gene *Phg*-*2* was identified in the cultivar Mexico 54 on chromosome Pv08 (Sartorato et al. 2000; Mahuku et al. 2011). Localization of reported markers revealed ALS resistance loci at the same region in Mesoamerican germplasm MAR 2 (Ferreira et al. 2000), Cornell 49-242 (Nietsche et al. 2000), Ouro Negro (Corrêa et al. 2001; Faleiro et al. 2003), G10474 (Mahuku et al. 2004) and G10909 (Mahuku et al. 2011). A genetic test for allelism with one pathotype indicated that the Mesoamerican line BAT 322 contained resistance locus *Phg*-2 as Mexico 54 (Namayanja et al. 2006), whereas in MAR 2, Mexico 54 and Ouro Negro, five additional independent dominant genes (*Phg*-*3* to *Phg*-*7*) were identified using different pathotypes (Caixeta et al. 2003, 2005; Sanglard et al. 2013). As for the Andean lines, several markers linked to ALS resistance have been reported (Table 1). *Phg*-*1* of the Andean cultivar AND 277 (Carvalho et al. 1998) was mapped to chromosome 1 (Goncalves-Vidigal et al. 2011). In the Andean accession G5686, Mahuku et al. (2009) identified a major resistance locus on Pv04 later confirmed by Oblessuc et al. (2012) and named ALS4.1^GS, UC^. In addition, Mahuku et al. (2009) reported two complementary resistance genes in G5686 on Pv09 (ALS9.1^GS^) and Pv04 (ALS4.2^GS^). Further QTL studies support a more quantitative nature of ALS resistance (Lopez et al. 2003; Teixeira et al. 2005; Mahuku et al. 2011; Oblessuc et al. 2012).

**Table 1:** Published simple sequence repeat (SSR), novel single nucleotide polymorphism (SNP) and high-resolution melting curve analysis (HRM, underlined) markers targeting previously reported angular leaf spot (ALS) resistance loci from Andean common bean (*Phaseolus vulgaris* L.) germplasm used in this study

Markers used in this study	Chromosome	Physical position (bp)	Annealing temperature (°C)	Forward primer sequences [SNP in brackets]	Reverse primer sequence	Marker linked with ALS resistance	Physical position of linked marker (bp)	Resistant parent (Andean)	Susceptible parent (Mesoamerican)	References
Marker21	1	6,443,388	63	ATGGCCAAAGCTTAGAATTAGAAGAT[A/C]	ACTCTTTGAGGGTGATATGATGGTAAG	BM146	6,303,308	Jalo EEP 558	Small white	Teixeira et al. (2005)
–	1	–		–	–	CV542014	50,513,706	AND 277	Ouro Negro	Goncalves-Vidigal et al. (2011)
Marker60	3	28,619,078	68	CAATCAAGCAAAGGTTGATGTAGAG[A/G]	TCCATGTTTACGATTGGACCTATGCA	PVBR106	21,259,940	CAL 143	IAC-UNA	Oblessuc et al. (2012)
Marker27	3	36,169,137	63	AACTTCCACATCTTTATTTGGACT[A/T]	GTTCGTATATTCTCTTTGCTACTGAAACT	BM159	38,679,369	CAL 143	IAC-UNA	Oblessuc et al. (2012)
Pvctt001^a^	4	514,757	48	GAGGGTGTTTCACTATTGTCACTGC	TTCATGGATGGTGGAGGAACAG	Pv-ctt001^a^	514,757	G5686	Sprite (Andean)	Mahuku et al. (2009)
						RGA 6	7,825,469	G19833	DOR364	Lopez et al. (2003)
Marker7	4	41,508,591	64	GTGTGATGGTGTTACTTCAAAAGAT[A/G]	GTGCAACCGTGACCACAATAAATCA	RGA14	41,880,058	G19833	DOR364	Lopez et al. (2003)
Marker8	4	42,310,722	66	CCCTGGCCAACCTAATGG[C/T]	AAATGGTGGTGGCTGCACTTAGC					
Marker48	4	43,325,042	62	ACACCAAGCCAAAATTAGAACAA[G/C]	GTAAATTAACTTCTAACTTGTTGCTTGGTC	Pv-atct001	44,019,142	ESAL 550	Carioca MG	Ferreira da Silva et al. (2003)
Marker63	4	43,497,706	60	GAAATGAGGCTAACAAGGAGGTT[A/G]	AGCCTGCAAGATTTTCAGATATCCT					
4M437	4	43,768,004	63	CACAGAACCAACAGTTTCTAAGC	GGGTGCAACCAAGAGTTTT					
Marker50	4	43,773,443	63	CCAGGTAAATAGGGTAATGAAGTTG[G/T]	ACTCATGAGATTGTGTATGGCCAACC	Pv-atgc002	44,019,876	CAL 143	IAC-UNA	Oblessuc et al. (2012)
4M439	4	43,915,434	61	TGTGGATCTCCACCTAGCAG	CTGCTCTTAGAACTTTGGAGATTC					
4M442	4	44,267,293	63	GAGGAAAATGCCCTTTAGCA	AGTGGACCCAAATGATGAGC	Pv-ag004^a^	44,019,894	G5686	Sprite (Andean)	Mahuku et al. (2009)iiiiii
Marker9	4	44,740,001	64	CCAAACAAATTCACACACCAAACT[A/C]	CTATGCATGACAATCTGTGAGTGAAAGG					
Marker31	5	38,366,776	68	TTCAACACCAAAGACATTCAAACTA[A/G]	GGTGTTCCTCATTTTCTGCTTCCTATT	Pv-at006	38,198,674	CAL 143	IAC-UNA	Oblessuc et al. (2012)
						Pv-br124	25,088,610	CAL 143	IAC-UNA	Oblessuc et al. (2012)
Marker13	7	36,334,463	70	TATTTACGCTCTAACCTGTTTGTATC[A/G]	TTGTCCCTTTTGTGAACTCAAGGCA	BM210	31,123,632	Jalo EEP 558	Small white	Teixeira et al. (2005)
MarkerJ2	8	58,385,592	62	TCCACATTCACATCTTCTTCAT[T/C]	TATACTCCTGCCACGCATTGA	PF13	45,959,995	G10909 (Meso)	Sprite (Andean)	Mahuku et al. (2011)
						SCARM02	45,959,567	Ouro Negro (Meso)	TO	Queiroz et al. (2004)
Marker32	9	14,793,993	63	TTCTCTGGGGAAAATGCATTG[C/T]	GACGTGTTTATATGCATTTGTCAATAGTCC	Pv-at007	16,738,017	G5686	Sprite (Andean)	Mahuku et al. (2009)
Marker33	9	17,264,951	65	CCACAGTCCCATTTCAGTCAG[A/G]	GTTTCTAGTGGTGAGTTTGTTGTTGTCA					
MarkerA1	10	7,151,110	62	CACTGCACTATATGCACATAAGA[A/G]	CAGTTCCCCAGAACATTAGCA	RGA9	3,815,638	G19833	DOR364	Lopez et al. (2003)
						RGA12	4,156,093			
						RGA7	8,784,684			
MarkerA4	10	9,729,049	62	TTTGAAGTGGATACATAATAGACCT[C/T]	TTTATCGGCATTCTGTGCAA	IAC137	4,893,842	CAL 143	IAC-UNA	Oblessuc et al. (2013)
Marker17	10	38,010,446	63	AGCAGCAGAATTCTGCAATC[G/T]	GGTTTTCTGGTTTTGGGTGGTAAATG	GATS11b (ATA220)	32,692,053	CAL 143	IAC-UNA	Oblessuc et al. (2013)

For the SNP markers genotyped by melting temperature (*T*_m_) shifts, the Tm shift was enhanced by a gcgggc or gcgggcagggcgg-tail added to the forward primer sequences for each SNP allele [in brackets] as described in Wang et al. (2005). Previously reported markers linked to ALS resistance and their physical positions are given along with the respective source of resistance and the corresponding reference

^a^SSR marker derived from Yu et al. (2000) evaluated by Mahuku et al. (2009) and in the current study

Wang et al. (2005) reported a genotyping method based on shifting melting temperatures (*T*_m_) of PCR amplicons introduced by allele-specific primers differing in *T*_m_. However, the primer design is rather inflexible due to the necessity for the primers to end exactly on the SNP. High-resolution melting curve analysis (HRM) developed by Gundry et al. (2003) and Wittwer et al. (2003) can overcome this disadvantage: Any sequence polymorphism between amplicons can be detected by fluorescence in much larger PCR amplicons (Reed and Wittwer 2004; Montgomery et al. 2007). HRM was used to fine-map resistance loci based on a well-defined DNA sequence polymorphism (Lehmensiek et al. 2008) or even when type, number and composition of the DNA sequence polymorphism in a particular PCR amplicon were unknown (Studer et al. 2009).

In this study, we aimed at (1) validating effective ALS resistance regions in common bean, (2) fine-mapping the source of resistance to identify candidate genes for ALS resistance and (3) developing closely linked markers for breeding applications.

## Materials and methods

### Plant material

A cross between the resistant Andean common bean genotype G5686 and the susceptible cultivar Sprite had been used for the development of an F2 population to characterize the genetics of ALS resistance (Mahuku et al. 2009). This population was now advanced to an F_3_ and F_4_ population with 180 and 153 individuals, respectively. Additionally, a resistant F_1_ plant backcrossed to the susceptible parental genotype was used to generate a BC1F_2_ mapping population (89 individuals). G5686 is a highly ALS-resistant Andean dry bean accession from Ecuador (Mahuku et al. 2009). Sprite is an Andean snap bean cultivar (Cunha et al. 2004; Gepts et al. 2008) and was susceptible to over 400 of 503 tested ALS isolates (Mahuku et al. 2009).

### Phenotypic evaluation of angular leaf spot (ALS) resistance

Plants were grown in the greenhouse for 17 days at 24–32 °C and infected with the *P. griseola* isolate 268-COL belonging to pathotype 31-0 (Pastor-Corrales et al. 1998; Mahuku et al. 2009). The inoculation with *P. griseola* was done according to the CIAT practical guide (Castellanos et al. 2011). The standard visual scale in which 1–3 means resistant, 4–6 intermediate and 7–9 susceptible was used for phenotypic scoring (Schoonhoven and Pastor-Corrales 1987). The plants were evaluated 11, 15 and 20 days after inoculation in the first cycle. Additionally, at the first evaluation, visible hypersensitive reaction (HR) was recorded. In a second infection cycle with 139 selected recombinant descendants, faster disease development was noted. Plants were visually scored after 9, 13 and 15 days post-inoculation. The calculated area under the disease progress curve (AUDPC) was used for classification into susceptible and resistant plants (Mahuku et al. 2009).

### Genotyping

Genotyping was conducted according to the strategy of Peleman et al. (2005): The 180 F_3_ plants were genotyped using two SSR and 17 *T*_m_ markers which were found to be polymorphic out of a set of 64 tested *T*_m_ markers (Table 1, Supplemental Table S1). The 153 F_4_, 89 BC_1_F_2_ and 139 F_4_/F_5_/BCF_3_ descendants were genotyped using three selected SNPs (Marker63, Marker50 or Marker9) in order to identify recombination events in a particular region of interest on chromosome Pv04. Finally, 47 recombinant plants were selected and genotyped using three HRM markers, which were found to be polymorphic out of 27 tested amplicons. Primers were designed by the Primer3web version 4.0.0 (Koressaar and Remm 2007; Untergasser et al. 2012).

DNA was extracted according to Xin et al. (2003) from approximately 30 mm^2^ young trifoliate leaf sample tissue or young unopened trifoliate leaves, using 100 µl buffer A (50 mM NaOH, 2 % Tween 20) and 75 µm buffer B (100 mM Tris–HCl, 1.7 mM EDTA, pH 7.3). The extract was diluted 1:10 in distilled water for PCR.

### Simple sequence repeat (SSR) genotyping

SSR markers Pv-ag004 and Pv-ctt001 from Yu et al. (2000) were amplified by PCR in a 20- µl reaction volume. The PCR mix contained 5 μl of genomic DNA solution, 1X Taq buffer [10 mM Tris–HCl pH 8.8, 50 mM KCl, 0.8 % (v/v) Nonidet P40 (Fermentas)], 2.5 mM MgCl_2_, 0.4 mM dNTPs mix (Promega), 0.2 μM of each primer (forward and reverse) and 0.15 μl of homemade Taq polymerase. 0.1 % Bovine serum albumin (BSA) and 1 % polyvinylpyrrolidone (PVP) were added to counteract polymerase inhibitors present in the DNA extract (Xin et al. 2003).

Pv-ag004 and Pv-ctt001 were amplified under the following PCR conditions: initial denaturation at 94 °C for 3 min followed by 35 cycles of denaturation at 94 °C for 30 s, 48 °C annealing temperature (Yu et al. 2000) for 30 s and extension at 72 °C for 8 min. The SSR marker visualization was described in Mahuku et al. (2009) with a separation at 80 V for approximately 1 h using an Owl T-Rex™ vertical S3S camera (ThermoFisher Scientific Inc, USA).

### Melting temperature (*T*_m_) shift genotyping

The physical positions of reported markers linked with ALS resistance were identified using sequences from the PhaseolusGenes Toolbox (http://phaseolusgenes.bioinformatics.ucdavis.edu) (2013) and positional information from the Phytozome platform (Goodstein et al. 2012). In those regions (Table 1), SNPs were selected out of a SNP collection provided by The Common Bean Coordinated Agricultural Project (BeanCAP) available on NCBI (2013) and Blair et al. (2013) presenting over 3,300 SNPs. SNP assays were designed following Wang et al. (2005). DNA was amplified by PCR in a total volume of 20 μl containing 4 μl of genomic DNA, 1X Taq buffer, 1.5 mM MgCl2, 0.2 mM dNTPs mix, 0.15 μM each primer (two allele-specific forward primers and the common reverse primer), 1X EvaGreen^®^ (Biotium) and 0.1 μl of homemade Taq polymerase. Amplification was carried out with the following program: initial denaturation at 94 °C for 3 min followed by 35 cycles of denaturation at 92 °C for 15 s, annealing of 15 s (temperature specific to each primer trio, Table 1) and extension at 72 °C for 15 s, finally followed by 10 min extension at 72 °C and 5 min at 10 °C. *T*_m_ shifts of the amplicons were measured by melting point analysis in a fluorescence-detecting thermocycler (Mx3000P Stratagene) and used to classify the samples into GG (homozygous DNA sequence of G5686), GS (heterozygous) or SS (homozygous DNA sequence of Sprite) genotypes.

### Genotyping using high-resolution melting curve analysis (HRM)

HRM genotyping was used to further delimit the position of the QTL ALS4.1^GS, UC^. Genes of common bean within this region were selected using the Phytozome genome browser (Phytozome.net 2014), and primer pairs spanning introns were designed with the Primer3web version 4.0.0 (Rozen and Skaletsky 2000). For genes without introns, EST sequences of different common bean genotypes were extracted from the database of Ramirez et al. (2005) and CleanEST (Lee and Shin 2009) and aligned to the common bean genome in order to identify SNPs. Primer pairs were designed to flank the SNPs in 150–300 bp distance. Primer sequences were compared against other closely related species such as soybean (*Glycine max*) or barrel medic (*Medicago truncatula*) using the Phytozome platform (Goodstein et al. 2012) to select most conserved primer sequences.

Thirteen EST-derived and fourteen intron-flanking primer pairs were designed between 43.5 and 44.5 Mbp on Pv04. The amplification was carried out in a PCR volume of 7.3 μl containing 1X LightScanner high sensitivity master mix (BioFire Diagnostics Inc., UT, USA) including LCGreen^®^ PLUS, 0.10 mM of each forward and reverse primer and 1.3 μl of DNA. Additionally, 14 μl of mineral oil was added to each sample, covering the mix to prevent evaporation during PCR and melting analysis. PCR conditions were set as following: denaturation for 2 min at 95 °C, 40 cycles of 30 s at 94 °C, 30 s annealing at the optimal temperature for each primer pair (Table 1) and 30 s for elongation at 72 °C followed by a final cycle of 2 min at 72 °C, 30 s at 94 °C and 30 s at 25 °C. Using a LightScanner Instrument (BioFire Diagnostics Inc.; 96-well plate format), the amplification product was melted ramping from 60 to 95 °C in 0.05 °C steps per second under continuous fluorescence measurement. The melting curves were related to genotype GG, SS and GS using the LightScanner^®^ and Call-IT^®^ software modules (BioFire Diagnostics Inc.).

### Identification of candidate genes within the region of QTL ALS4.1^GS, UC^

In order to identify homologies to previously reported candidate resistance genes, an NCBI megaBLAST query (Zhang et al. 2000) was conducted using the sequence in the region cosegregating with ALS4.1 of the common bean reference genome (Andean accession G19833, Schmutz et al. 2014). Function of genes analogous to Arabidopsis was studied using The Arabidopsis Information Resource (TAIR) (Swarbreck et al. 2008). Phylogenetic analysis of candidate genes and related common bean genes was carried out using the amino acid sequences of the candidate protein kinases. First, the sequences of the candidate kinases were used for a protein BLAST against the reference genome, collecting the first 100 hits of each candidate sequence. All sequences were then aligned using Clustal Omega (Sievers et al. 2011) and the results analyzed with the software package Mega5 for a comparative analysis and the construction of a maximum-likelihood tree (Tamura et al. 2011).

### Statistical analysis

Single and multiple QTL analyses were carried out using the R package *qtl* developed by Broman et al. (2003). Single QTL analysis and LOD score calculation were done by marker regression and standard interval mapping (200,000 bp steps, 1,000 permutations, 0.01 assumed genotyping error rate) in order to analyze the major QTL. *p* values were derived by the analysis of variance (ANOVA) if the residues showed normal distribution and using *lmPerm* R package of Wheeler (2010) if this was not the case. A multiple QTL model was built using the function stepwise with 200,000 bp steps, 1,000 permutations, 0.01 assumed genotyping error rate and 256 imputations in order to calculate a penalized LOD score, followed by analyses omitting one QTL at a time to obtain an ANOVA table.

## Results

### Phenotypic evaluation for angular leaf spot (ALS) resistance

In order to validate QTL reported in various Andean germplasm, 180 F_3_ (65 families) of the Sprite × G5686 population were evaluated with the *P. griseola* pathotype 31-0. Plants were classified into 119 resistant and 61 susceptible plants, showing AUDPC resistance values of <20 and ≥20, respectively. The ratio between the resistant and the susceptible plants is in accordance with one dominant resistance gene segregating in an expected 5:3 ratio, assuming independent F_3_ individuals (observed Chi-squared = 1.001, *p* = 0.317). HR was observed in 18 out of the 180 F_3_ plants. Hypersensitive plants were healthy and vigorous, showing only bright green dots of few millimeters of diameter on the leaf surface without damage to the leaf tissue.

### Multiple QTL analysis

Genotyping of these 180 F_3_ plants with 2 SSR and 20 SNP markers (Table 1) and subsequent multiple QTL analyses identified one major and two minor QTL explaining in total 80.1 % of the phenotypic variation for ALS resistance (Table 2). The major QTL (ALS4.1^GS, UC^), closely linked to Marker50 localized on chromosome Pv04 at around 43.7 Mbp, explained 75.3 % of the ALS resistance. Two smaller QTL (ALS10.1^DG, UC, GS^ and ALS9.1^GS^) explained 4.9 and 1.7 % of the ALS resistance, respectively. Out of three markers genotyped on Pv10, Marker17, localized at around 38 Mbp, was best linked to ALS10.1. As for ALS9.1, evaluated with two markers, it was most closely linked to Marker33 localized on Pv09 at around 17.2 Mbp.

**Table 2:** Multiple QTL analyses resulted in three significant QTL for angular leaf spot (ALS) resistance on chromosomes Pv04, Pv09 and Pv010. QTL models were designed using the stepwise function of R package *qtl* (Broman et al. 2003)

QTL model	Closest marker	Chromosome	Physical position (bp)	LOD	Explained variance (%)	*p* value (F)	Germplasm in which colocating QTL was identified
ALS4.1^GS, UC^: ALS10.11^DG, UC, GS^ + ALS9.1^GS^	–	4:10 + 9	–	63.0	80.1	0***	
ALS4.1^GS, UC^	Marker50	4	43,773,443	61.1	75.3	<2e−16***	G5686, ESAL 550^a^, G19833^b^, CAL 143^c^
ALS10.1^DG, UC, GS^	Marker17	10	38,010,446	8.7	5.0	1.17e−06***	G5686^d^, G19833^b^, CAL 143^c^
ALS4.1^GS, UC^: ALS10.1^DG, UC, GS^	–	4:10	–	5.8	3.2	4.31e−05***	–
ALS9.1^GS^	Marker33	9	17,264,951	3.2	1.7	0.00106**	G5686^d^
ALS5.2^UC, GS^	Marker31	5	38,198,674	1.5	3.7	<0.05 (permutation)*	G5686, CAL 143^c^

LOD score, percentage of explained variance and *p* value were derived from F statistics by dropping one QTL at a time resulting in the ANOVA table. An additional QTL (ALS5.2^UC, GS^) was identified by marker regression on Pv05

Significance levels: * *p* < 0.05, ** *p* < 0.01, *** *p* < 0.001

^a^Ferreira da Silva et al.(2003), ^b^Lopez et al. (2003), ^c^Oblessuc et al. (2012) and ^d^Mahuku et al. (2009) reported QTL at the same region in referred Andean germplasm

### Single QTL analysis

**Fig. 1:**
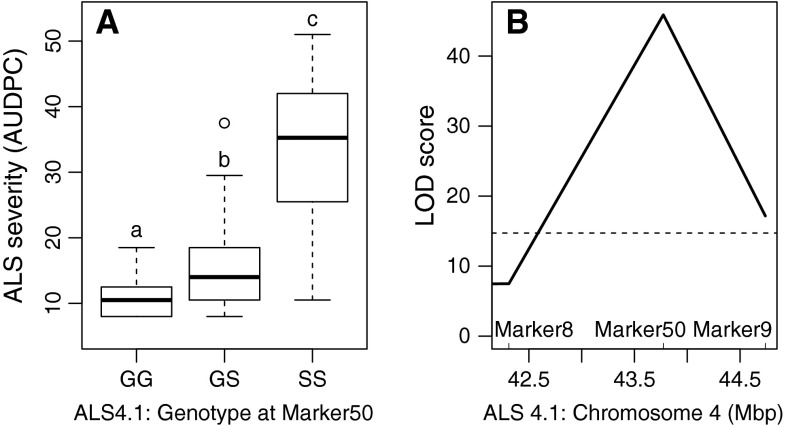
One major QTL controls angular leaf spot (ALS) resistance in the G5686 × Sprite population in an evaluation of 180 F_3_ plants infected with *Pseudocercospora griseola* (Sacc.) Crous and U. Braun pathotype (race 31-0). **a** *Box plot* sorted by the genotypes at Marker50 shows significant correlation (permutation *p* < 2e−16, 161 observations) to ALS disease scores, explaining 70.4 % of the phenotypic variation for ALS resistance. The *horizontal bar* (*bold*) indicates the median, the *box* represents inter-quartile range, *discontinuous lines* represent the upper and lower quartile, and outlier samples (>1.5 × inter-quartile range) are depicted by a *circle*. *Letters* indicate significant differences between genotypes using permutation tests. **b** LOD curve with Marker8, 50 and 9 revealed Marker50 at 43,773,443 bp as closest linked with ALS resistance gene. *Dashed line* represents LOD score threshold for the 5 % significance level

Four markers (Marker7, 8, 50 and 9) were analyzed in the region of ALS4.1. Single QTL analysis by interval mapping showed that Marker50 had the most significant effect on ALS resistance (LOD score of 45.9, Fig. 1a), masking all other QTL effects in the single QTL analysis. In accordance with the reported dominant inheritance of ALS resistance, the Marker50 genotypes GG and GS resulted in resistant phenotypes and SS in susceptible phenotypes (permutation *p* < 2e−16 including 172 observations, Fig. 1a). However, heterozygous GS genotypes had lower resistance levels indicating some codominance effect. Marker50 showed complete linkage with ALS resistance in the sense of all homozygous plants with genotype GG being resistant. Only three plants with a Sprite SS genotype on this locus (according to all four markers in the region) were evaluated as resistant, suggesting phenotypic escapes or involvement of other loci.

**Fig. 2:**
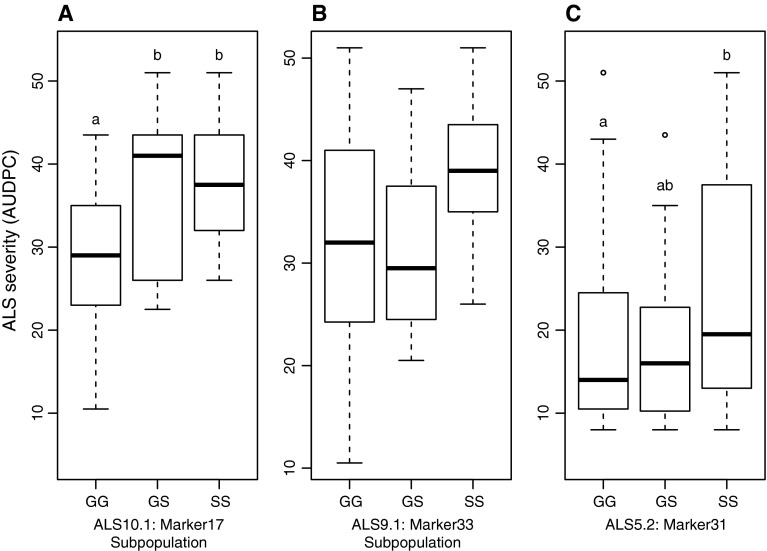
Effects of QTLs ALS10.1^DG, UC, GS^, ALS9.1^GS^ and ALS5.2^UC, GS^ on angular leaf spot (ALS) resistance in the G5686 **×** Sprite population infected with *Pseudocercospora griseola* (Sacc.) Crous and U. Braun pathotype (race 31-0). **a** Marker17 (ALS10.1) localized on chromosome Pv010 showed significant correlation with ALS resistance (*p* = 0.003, 52 observations) within the subpopulation of the F_3_ G5686 × Sprite population with homozygous SS genotypes at Marker50 (ALS4.1^GS, UC^). **b** In the same subpopulation, Marker33 (ALS9.1) on Pv09 showed a weak correlation with ALS resistance (*p* = 0.062, 47 observations). **c** Marker31 (ALS5.2) localized on Pv05 showed significant influence on ALS resistance (permutation *p* < 0.05, 130 observations) evaluating the whole F3 population. For each genotype, the *horizontal bar* (*bold*) indicates the median, the *box* represents inter-quartile range, *discontinuous lines* represent the upper and lower quartile, and outlier samples (>1.5 × inter-quartile range) are depicted by a *circle*. *Letters* indicate significant differences between genotypes using TukeyHSD (Marker17) and permutation tests (Marker31)

Marker17, linked to ALS10.1 localized on chromosome Pv010 at around 38.01 Mbp, showed weak linkage with ALS resistance (permutation *p* < 0.1, 169 observations). Considering only plants with homozygous SS genotypes at Marker50, correlation of Marker17 with ALS resistance was significant (*p* = 0.003 including 52 observations, Fig. 2a). Marker17 explained 18.2 % of phenotypic variation in those selected plants. Analysis of further markers at the beginning of Pv010, MarkerA1 (*p* = 0.010, 40 observations) and MarkerA4 (*p* = 0.044, 40 observations) at 7.15 and 9.73 Mbp, respectively, also resulted in a weak but significant correlation with ALS resistance considering only susceptible SS genotypes at Marker50.

Marker33 (permutation *p* < 0.1, 153 observations), linked to ALS9.1 on Pv09, only had a significant effect on ALS resistance in multiple QTL analysis together with Marker32 (permutation *p* < 0.2, 148 observations), positioned 2.5 Mbp upstream. In the subpopulation considering only plants with SS genotype of Marker50, Marker33 contributed weakly to ALS resistance (*p* = 0.062, 47 observations, Fig. 2b).

Marker31 (QTL ALS5.2^UC, GS^) on Pv05 explained 3.7 % of ALS resistance (permutation *p* < 0.05, 130 observations, Fig. 2c). However, QTL analysis resulted in a LOD score (1.5) below the calculated significance threshold requiring a LOD score above 14.5 according to a 5 % significance level. It was the only marker on Pv05 and could therefore not be included in multiple QTL analysis.

### Fine-mapping of the major QTL for ALS resistance

**Fig. 3:**
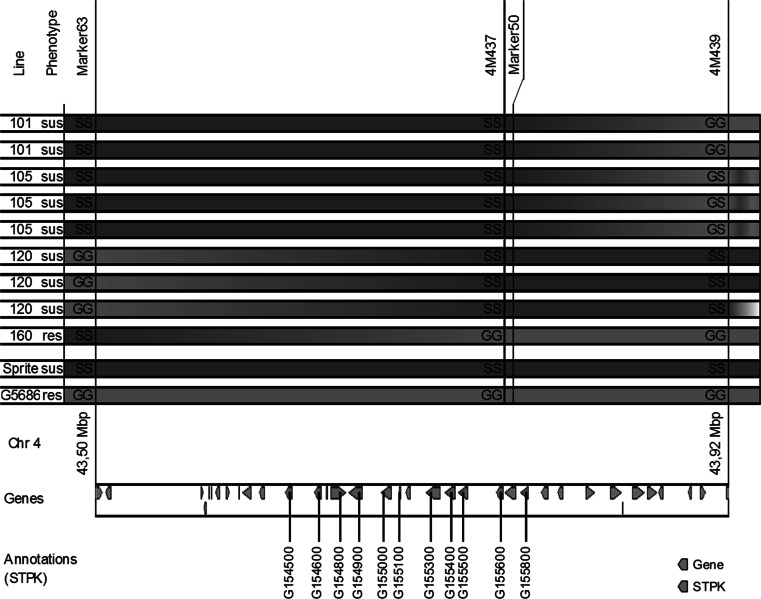
Fine-mapping of QTL ALS4.1^GS, UC^ for angular leaf spot (ALS) resistance in the G5686 × Sprite mapping population points to a locus harboring repetitive serine/threonine protein kinases (STPK). Correlations of Sprite genotypes (SS) and G5686 genotypes (GG) with the susceptible (sus) and resistant (res) phenotypes of selected informative recombinant plants. Analysis of recombinants between flanking Marker63 and 4M439 mapped ALS4.1 to a 417,728-bp region. Shown below, 36 genes within the delimited ALS4.1 region that are annotated in the reference sequence. The delimited ALS4.1 region harbors 11 STPKs containing leucine-rich repeats (Schmutz et al. 2014)

Fine-mapping of the ALS4.1 region was conducted on 47 recombinants out of 561 screened plants (selected from 180 F_3_, 153 F_4_, 89 BC_1_F_2_ and 139 F_4_/F_5_/BC_1_F_3_ descendants that were investigated for recombination events in the genomic region of ALS4.1) using seven polymorphic markers: four SNP markers (Marker8, Marker63, Marker50 and Marker9) and three HRM markers (4M437, 4M439 and 4M442). Homozygous plants at 4M437 showed no recombination between the marker and the resistance gene, whereas the flanking Marker63 identified four and 4M439 two recombinant plants (Fig. 3). Hence, the region of ALS4.1 was delimited by the markers Marker63 and 4M439, extending from 43,497,706 to 43,915,434 bp.

### Toward marker-assisted selection

To make this resistance locus available for marker-assisted selection (MAS) to breeding programs, which do not have SNP genotyping facilities, we set up two assays at this locus for outsourcing at the commercial provider LGC genomics (Hertfordshire, UK, http://www.lgcgenomics.com). Assay MAS_ALS4a is based on the polymorphism from Marker50 (Chr04, pos_43,773,443_T/G) and assay MAS_ALS4b (Chr04, pos_43,794,830_T/C) may have a higher specificity, as it was identified based on the recent whole-genome resequencing of genotype G5686 as a unique polymorphism compared with eight other resequenced genotypes (AFR298, G10474, SEA5, MDE23-24, AND696, G40001, G35346 and VAX1, unpublished data). Genotypic data in Table 3 suggest using MAS_ALS4b for marker-assisted selection.

**Table 3:** Specificity of markers tagging QTL ALS4.1^GS, UC^ evaluated on a panel of Andean and Mesoamerican genotypes, comparing Marker50 and genotyping data obtained from LGC Genomics genotyping facility MAS_ALS4a and MAS_ALS4b. Comparison of marker50 and MAS_ALS4a (based on same SNP) shows that MAS_ALS4a assay design failed, which is moderately unusual. Sprite allele is not called, whereas G5686 and heterozygous samples are called as G5686 allele. Assay MAS_ALS4b is most specific, distinguishing G5686 and AND277 from the other genotypes

Genotype	Marker ID		
	MAS_ALS4a	Marker50	MAS_ALS4b
G5686	G:G	G:G	C:C
SPRITE	?	T:T	T:T
G10474	?	?	T:T
AND 277	G:G	G:G	C:C
BAT 93	?	T:T	T:T
G855	?	T:T	T:T
G1805	?	T:T	T:T
G5653	?	T:T	T:T
G10909	G:G	G:G	T:T
G14519	?	T:T	T:T
G18970	?	T:T	T:T
G40001	?	T:T	?
G23823E	?	T:T	T:T
G4691	?	T:T	T:T
JULES	?	T:T	T:T
MBC 7	G:G	G:G	T:T
MBC 39	G:G	G:G	T:T
MEXICO 54	?	T:T	T:T
NUA 56	?	T:T	T:T
VAX 1	?	T:T	T:T
VAX 6	?	T:T	T:T
XAN 112	?	T:T	T:T

### Identification of putative resistance genes within ALS4.1^GS, UC^

According to the reference sequence of the Andean common bean accession G19833, 36 genes are predicted in the region of the ALS4.1. Resequencing data did not reveal any polymorphisms in the coding sequences of the candidate genes (unpublished data), pointing toward undetected insertions. Among the 36 genes, 11 very closely related to serine/threonine protein kinases (STPK) are annotated (Fig. 3). BLAST searches revealed two *Arabidopsis thaliana* (At)-like STPKs described as putative resistance genes: Probable LRR receptor-like At1g07560 and At1g18390 involved in response to chitin and respiratory burst defense, best matching common bean genes Phvul.004G154600 and Phvul.004G154800, respectively (Phytozome.net 2014). To further characterize the candidate kinases, the amino acid sequence of Phvul.004G155000 was used for a BLAST search to identify related genes in the bean genome. The amino acid sequences of the first 100 hits were aligned with the candidate STPKs, and a maximum-likelihood tree was built. Eight of the 11 kinases are part of a subfamily, together with other two kinases from Pv02 and 10, and are most related to each other, suggesting that they originate from recent local duplications. The other three kinases were grouped apart and are more closely related to kinases present in Pv07, Pv03 and Pv02. (Supplemental Figure S1). Predicted functions of homologs in plant defense and the repetitive nature make these kinases prime candidates to cause the observed resistance ALS.

## Discussion

### Characterization of a major resistance locus

Phenotypic evaluation of an F_3_ mapping population derived from a resistant Andean common bean genotype G5686 and a susceptible cultivar Sprite for ALS resistance demonstrated the presence of one dominant major resistance locus on chromosome Pv04 and three minor loci. Molecular characterization and multiple QTL analyses confirmed a major resistance source (ALS4.1^GS, UC^) explaining 75.3 % of the phenotypic variation for ALS resistance, which was previously tagged with the SSR marker Pv-ag004 (Mahuku et al. 2009). In the present study, ALS4.1 was delimited to a region between 43,497,706 and 43,915,434 bp on Pv04 and tagged with two markers linked to ALS resistance. The two markers, HRM marker 4M437 and *T*_m_ shift Marker50, were developed based on EST sequence alignments (Ramirez et al. 2005; Lee and Shin 2009) or SNP collections (NCBI 2013; Blair et al. 2013), respectively. This approach enabled the development of markers at any genomic region, independent of polymorphic microsatellite motifs as necessary for SSR markers. In combination with the HRM technology, allowing for fast and accurate closed-tube genotyping in any genetic background, the markers presented here enable efficient marker-assisted introgression of ALS4.1 into advanced breeding germplasm of common bean. The value of G5686 as an Andean source of resistance to withstand both Andean and Mesoamerican pathotypes of *P. griseola* has been recognized in previous studies (Pastor-Corrales et al. 1998; Mahuku et al. 2009). Ferreira da Silva et al. (2003) reported marker PV-atct001 linked to a resistant allele in a cross of ESAL 550 and Carioca MG. Lopez et al. (2003) reported a QTL near RGA14 in DOR364 × G19833. Now available physical marker positions suggest these may be the same QTL ALS4.1^EC, DG, GS, UC^ which remains to be confirmed. The ALS resistance gene in ALS4.1 is likely to be one of the genes *Phg*-*2*, *Phg*-*3* or *Phg*-*4,* reported in AND277 by Caixeta et al. (2005). But as these are not mapped and resistance evaluations used other isolates, further allelism tests are required to identify which gene exactly is underlying ALS4.1

### ALS4.1^GS, UC^ embeds a repetitive genome region including potential resistance genes

According to the common bean reference sequence, the ALS4.1 region harbors among its 36 genes repetitive homologs of putative disease resistance genes (Schmutz et al. 2014). Similar observations were reported for other major resistance loci, as, for example, the *Mla* locus conferring resistance to powdery mildew in barley (*Hordeum vulgare* L.), where 15 out of 32 annotated protein-coding genes can be associated with plant defense responses (Wei et al. 2002). In the present study, 11 annotated genes containing STPK domains were identified in the region of ALS4.1, eight and three genes, respectively, in two phylogenetically closely related groups. This is interesting since plant resistance genes are often clustered in the genome and evolve rapidly via diverse mechanisms (Smith et al. 2004; David et al. 2009). For example, resistance against soybean cyst nematode is mediated by copy number variation of a 31-kbp genome segment fortifying the expression of several dissimilar genes in a repetitive multigene region (Cook et al. 2012). STPKs phosphorylate hydroxyl groups of serine or threonine residues which is essential for various signaling pathways in eukaryotes including pathogen-triggered immunity (Park et al. 2011; Zhang et al. 2013). For example, the *Pto* protein in tomato containing a STPK domain (Martin et al. 1993; Loh et al. 1998) induces HR by recognizing two specific *Pseudomonas syringae* pathogen effectors (Dong et al. 2009). Similar STPK-based defenses were reported in Arabidopsis (Warren et al. 1999; Swiderski and Innes 2001) and wheat against powdery mildew (Cao et al. 2011). The involvement of STPKs in pathogen response and the repetitive arrangement also found at other previously described resistance loci (e.g., Vallejos et al. 2006) make this STPK cluster a likely candidate to cause the observed ALS resistance. Resequencing data of G5686 did not reveal any polymorphisms to the reference G19833 that may cause the resistance. While this may be explained with the difficult assembly of this region due to its various local duplications, we hypothesize the G5686 likely contains additional duplications. To detect new genes, a de novo assembly necessitating deeper sequencing data is required.

### Hypersensitive reaction is probably linked with ALS4.1^GS, UC^ and ALS10.1^DG, UC, GS^

Plants usually respond to biotrophic fungi such as *P. griseola* at early infection stages with HR (Glazebrook 2005). The At1g18390 gene, sharing 54.3 % protein sequence homology with STPK Phvul.004G154800 in ALS4.1, is likely involved in respiratory burst within the HR (Phytozome.net 2014; TAIR 2013). Indeed, ALS4.1 was linked with observed HR as 16 out of 18 plants (observed *χ* ^2^= 5.348, *p* = 0. 021) showing HR also had GG genotype at Marker50 within ALS4.1. Interestingly, Marker17 linked to ALS10.1 was also associated with HR as 15 out of 17 plants (1 missing data, observed *χ* ^2 ^= 4.804, *p* = 0.028) showed both HR and GG genotype at Marker17, possibly explaining the small but significant resistance improvement when both QTL present. This is in line with Zhou et al. (1995), who observed enhanced HR in the presence of additional genes besides resistance genes *Pto* and *Prf*. The fact that HR was observed only in 18 out of 180 plants of the F_3_ population indicates that HR may require several genes (Zhou et al. 1995; Salmeron et al. 1996), a low phenotypic penetrance, or that activation of systemic acquired resistance (Oh and Martin 2011) prevented the plants from visible symptoms.

### ALS4.1^GS, UC^ is a major QTL suitable for marker-assisted introgression of ALS resistance

In a previous study, three dominant and complementary genes linked with SSR markers Pv-ag004 (ALS4.1 region), Pv-at007 (ALS10.1 region) and Pv-ctt001 (beginning of Pv04), respectively, were reported to confer ALS resistance in an F_2_ population of the same cross infected with the same *P. griseola* pathotype (Mahuku et al. 2009). The authors concluded the involvement of three dominant and complementary genes, statistically expecting 56.3 % of plants which carry a G allele at Pv-ag004 (ALS4.1) to be resistant to ALS, whereas the observed proportion was 68 %. In contrast, in the current study, 89 % of plants with a G allele at Pv-ag004 were resistant to ALS, and even 97 % of the plants with the homozygous genotype GG (data not shown). These percentages depend on the classification of heterozygous plants to be resistant or susceptible, which might have been different in Mahuku et al. (2009) and confounded the effect of Pv-ag004. Moreover, environmental conditions might have affected the otherwise identical phenotyping procedure. In the current study, ALS4.1 was masking the minor effect of Pv-at007 linked to ALS9.1^GS^ and an effect of Pv-ctt001 could not be detected (permutation *p* > 0.5 including 78 observations). Consistent detection of ALS4.1 across repeated experiments explaining the major proportion of the observed resistance rather indicates that ALS resistance in this population is controlled by one independent major locus modified by few minor QTL. We conclude that ALS4.1 has a high value as a source for marker-assisted introgression of ALS resistance, with polymorphism T/C on Chr04 at 43,794,830 bp (marker MAS_ALS4b) showing highest specificity for G5686.

### Various QTL control ALS resistance

ALS resistance loci in the region of ALS4.1 were reported in different germplasm screened with several pathotypes (Faleiro et al. 2003; Lopez et al. 2003; Caixeta et al. 2005; Mahuku et al. 2011) and in different environments (Oblessuc et al. 2012). For example, Ferreira da Silva et al. (2003) identified a major ALS resistance locus in the same region as ALS4.1 evaluating the ESAL 550 cultivar in a field experiment. Lopez et al. (2003) and Oblessuc et al. (2012) reported QTL in the same region explaining a smaller part of the variation using various Andean genotypes and experimental conditions. Oblessuc et al. (2012) named two closely adjacent QTL on Pv04, ALS4.1 and ALS4.2, which encase the QTL reported here. Hence, we hypothesize that these are actually one QTL named ALS4.1^GS, UC^. AND277, the only genotype to share the G5686 allele for the MAS_ALS4b marker in the evaluated set (Table 1), is similar but not identical to G5686 (correlation 0.88 based on 650 markers, unpublished data). Also available pedigree information does not indicate G5686 in the ancestry of AND277; hence, there is no clear evidence that G5686 and AND277 carry the same allele. On Pv10, Oblessuc et al. (2012) identified the major QTL ALS10.1^DG, UC, GS^ (Marker17) explaining over 20 % of ALS resistance in the field, in both dry and wet season using CAL 143 as a source of resistance (Oblessuc et al. 2012). ALS9.1^GS^ was previously described by Mahuku et al. (2009) but not named. Taken together, the significant QTL in this study was also found in other experiments and genotypes, supporting the findings in this work.

ALS resistance initially reported to be monogenetic evaluating only one pathotype (Ferreira et al. 2000; Corrêa et al. 2001) was shown to be quantitatively inherited evaluating different pathotypes (Faleiro et al. 2003; Caixeta et al. 2005). Pathotype-specific resistance in common bean was also reported considering different pathotypes of rust (Park et al. 1999; Faleiro et al. 2003) and anthracnose (Faleiro et al. 2003; Rodriguez-Suarez et al. 2007). Since G5686 proved resistance against a wide range of pathotypes (Mahuku et al. 2009), it appears likely that minor QTL or additional QTL will add specific resistance to other pathotypes.

## Conclusion

A major QTL explaining 75.3 % of ALS resistance in the G5686 × Sprite population was validated, mapped to 418 kbp on chromosome Pv04 and tagged with two closely linked SNP markers (Marker50 and 4M437) allowing efficient MAS. ALS4.1^GS, UC^ defines a region of 36 genes including 11 STPKs, which are likely candidates for the resistance gene. Additionally, three minor QTLs were identified. The Andean resistance loci ALS4.1 and *Phg*-*1* as well as the Mesoamerican *Phg*-*2* can now be combined and tested in elite cultivars in order to pyramid resistance genes.

### **Author contribution statement**

BR, CJ, CM, BS and BK conceived and designed the study, and BK performed the experiments. BR, CJ and BS contributed with material, reagents and analysis tools, while BK, BR, JDL and CM helped analyzing the data. The manuscript was drafted by BK and further improved by CM, BR and BS. All authors read and approved the manuscript for publication.

## Electronic supplementary material

Below is the link to the electronic supplementary material.

Figure S1 Phylogenetic analysis of common bean kinases most related to those found at QTL ALS4.1^GS, UC^. BLAST searches of all 11 serine/threonine protein kinases (STPK) within ALS4.1, marked in yellow, were carried out to identify most related STPKs in the bean reference genome (Schmutz et al. 2014). STPKs in ALS4.1 fall into two groups of highly related genes, indicating local duplications (PDF 22 kb)

Table S1 Primer sequences Names of polymorphic markers, single nucleotide polymorphism (SNP) ID, sequences and physical position of all used primers are listed. (XLSX 23 kb)
