# Supplementary figures and images for: Fine-mapping of a major QTL controlling angular leaf spot resistance in common bean (Phaseolus vulgaris L.)
Source: Theor Appl Genet. 2015 Mar 5;128(5):813–26. doi: 10.1007/s00122-015-2472-6 (PMC4544502; doi:10.1007/s00122-015-2472-6)

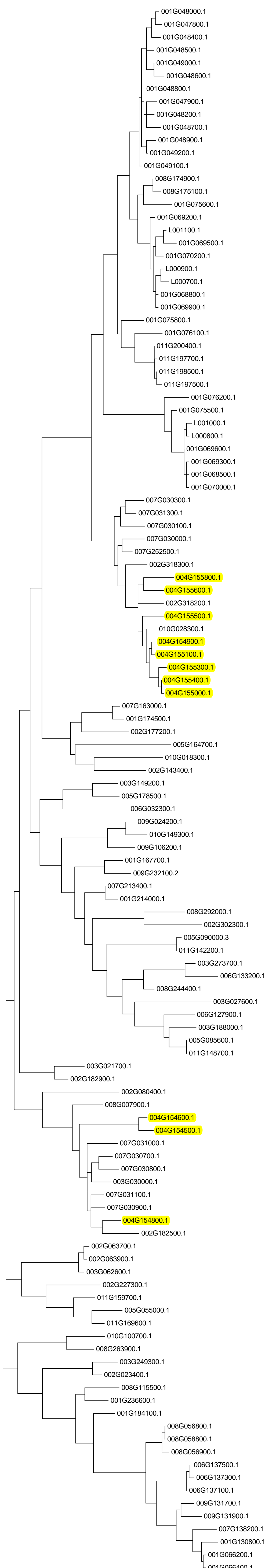

Supplement: Supplementary file 1 — Figure S1 Phylogenetic analysis of common bean kinases most related to those found at QTL ALS4.1GS, UC. BLAST searches of all 11 serine/threonine protein kinases (STPK) within ALS4.1, marked in yellow, were carried out to identify most related STPKs in the bean reference genome (Schmutz et al. 2014). STPKs in ALS4.1 fall into two groups of highly related genes, indicating local duplications (PDF 22 kb) [file 122_2015_2472_MOESM1_ESM.pdf]
